# Supplementary material for: Muscular and Kinematic Responses to Unexpected Translational Balance Perturbation: A Pilot Study in Healthy Young Adults
Source: Bioengineering (Basel). 2023 Jul 13;10(7):831. doi: 10.3390/bioengineering10070831 (PMC10376184; doi:10.3390/bioengineering10070831)
Supplement: Supplementary file 1 [file bioengineering-10-00831-s001.zip › bioengineering-2443331-supplementary.pdf]

bioengineering

MDPI

Supplementary Materials

Muscular and Kinematic Responses to Unexpected Translational Balance Perturbation: A Pilot Study in Healthy Young Adults

Cheuk Ying Tong [1,†], Ringo Tang-Long Zhu [1,2,†], Yan To Ling [1,3], Eduardo Mendonça Scheeren [4], Freddy Man Hin Lam [5], Hong Fu [6,*], and Christina Zong-Hao Ma [1,2,*]

[1] Department of Biomedical Engineering, The Hong Kong Polytechnic University, Hong Kong SAR 999077, China; cheuk-ying.tong@connect.polyu.hk (C.Y.T.), ringo-tanglong.zhu@connect.polyu.hk (R.T.-L.Z.), jane.yt.ling@connect.polyu.hk (Y.T.L.)

[2] Research Institute for Smart Ageing, The Hong Kong Polytechnic University, Hong Kong SAR 999077, China

[3] Centre for Developmental Neurobiology, King's College London, London SE1 1UL, UK

[4] Graduate Program in Health Technology, Pontifícia Universidade Católica do Paraná, Curitiba 80215-901, Brazil; eduardo.scheeren@pucpr.br (E.M.S.)

[5] Department of Rehabilitation Sciences, The Hong Kong Polytechnic University, Hong Kong SAR 999077, China; freddy-mh.lam@polyu.edu.hk (F.M.H.L.)

[6] Department of Mathematics and Information Technology, The Education University of Hong Kong, Hong Kong SAR 999077, China; hfu@eduhk.hk (H.F.)

* Correspondence: czh.ma@polyu.edu.hk (C.Z.-H.M.); hfu@eduhk.hk (H.F.); Tel.: +852-2766-7671 (C.Z.-H.M.); Fax: +852-2334-2429 (C.Z.-H.M.).

† These authors contributed equally to this work.

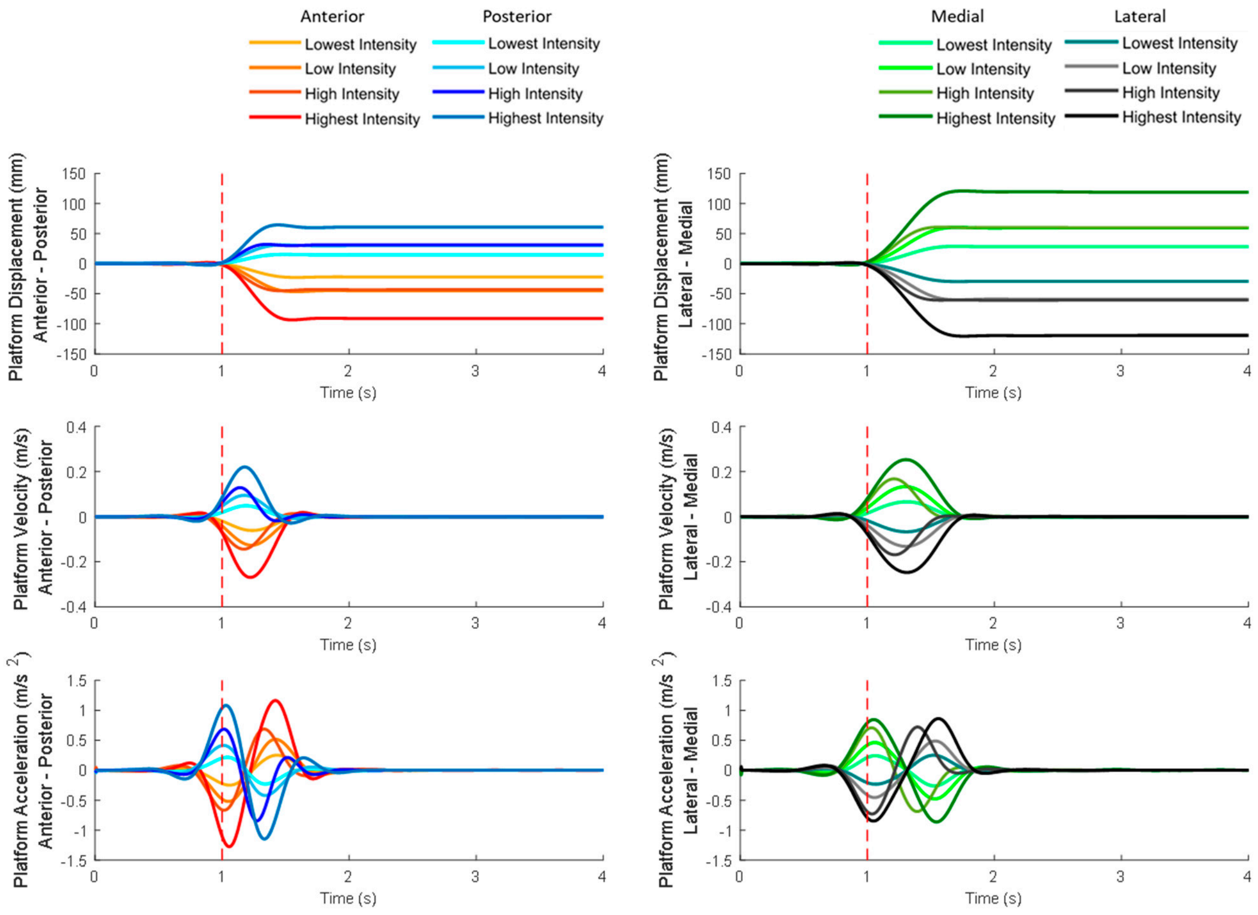

Figure S1. The mean platform displacements, velocities, and accelerations of twelve subjects following the unexpected anterior, posterior, medial, and lateral perturbations with four intensities (*n* = 12). (Note: **Red dotted line** specifies the start of the balance perturbation.).

Table S1. Mean and ICC values of pulling parameters examining the reliability of the moving-platform balance perturbation system (*n* = 12).

		**Pulling Duration (s)**		**Max. Displacement of Platform (cm)**		**Normalized Max. Displacement of Platform (% Height)**		**Max. Velocity of Platform (m/s)**		**Max. Acceleration of Platform (m/s²)**	
**Direction**	**Intensity**	**Mean**	**ICC**	**Mean**	**ICC**	**Mean**	**ICC**	**Mean**	**ICC**	**Mean**	**ICC**
Anterior	Highest	0.379	0.989*	9.4	0.859*	5.5	0.729*	0.27	0.930*	1.28	0.944*
	High	0.245	0.983*	4.5	0.968*	2.7	0.965*	0.14	0.981*	0.69	0.991*
	Low	0.359	0.979*	4.7	0.712*	2.7	0.703*	0.13	0.943*	0.53	0.987*
	Lowest	0.350	0.866*	2.3	0.919*	1.3	0.919*	0.06	0.934*	0.26	0.976*
Posterior	Highest	0.251	0.976*	6.5	0.716*	3.8	0.565*	0.22	0.849*	1.10	0.952*
	High	0.162	0.954*	3.4	0.631*	2.0	0.499*	0.13	0.822*	0.74	0.967*
	Low	0.238	0.979*	3.1	0.955*	1.8	0.936*	0.09	0.981*	0.43	0.994*
	Lowest	0.231	0.977*	1.5	0.268*	0.9	0.493*	0.05	0.971*	0.24	0.992*
Medial	Highest	0.507	0.995*	12.1	0.895*	7.1	0.703*	0.25	0.933*	0.85	0.971*
	High	0.328	0.976*	6.1	0.888*	3.6	0.213*	0.17	0.817*	0.72	0.988*
	Low	0.480	0.995*	6.0	0.725*	3.5	0.394*	0.13	0.913*	0.47	0.978*
	Lowest	0.466	0.992*	2.9	0.651*	1.7	0.535*	0.07	0.855*	0.25	0.958*
Lateral	Highest	0.440	1.000*	12.1	0.800*	7.1	0.521*	0.25	0.539*	0.86	0.949*
	High	0.472	0.999*	6.2	0.807*	3.6	0.620*	0.17	0.628*	0.74	0.973*
	Low	0.434	1.000*	6.0	0.912*	3.5	0.696*	0.13	0.897*	0.46	0.968*
	Lowest	0.434	0.999*	3.0	0.886*	1.7	0.718*	0.07	0.774*	0.24	0.944*

Note: The intraclass correlation coefficient (ICC) of three perturbations with the same direction and intensity in 12 subjects was calculated. Max.: Maximal. * Significant difference existed in the intraclass correlation coefficient test ($p < 0.05$).
